# How Kirschner Wires Crossing Each Other at the Fracture Site Affect Radiological and Clinical Results in Children With Gartland Type 3 Supracondylar Humerus Fractures?

**DOI:** 10.7759/cureus.50919

**Published:** 2023-12-21

**Authors:** Bulent Kilic, Ozcan Kaya, Mustafa Caliskan, Deniz Gulabi

**Affiliations:** 1 Orthopaedics and Traumatology Department, Saglik Bilimleri University, Kanuni Sultan Süleyman Training and Research Hospital, Istanbul, TUR; 2 Orthopaedics and Traumatology Department, Marmara University Faculty of Medicine, Istanbul, TUR

**Keywords:** crossed-pin fixation, gartland type 3 fractures, fracture line, closed reduction, supracondylar humerus fracture

## Abstract

Background

In this study, we compared two groups of children with Gartland Type 3 supracondylar humerus fractures with respect to the crossing point of Kirschner wires (K-wires) in terms of radiological and clinical results after closed reduction and fixation with the crossed-pin technique. We hypothesized that even if medial and lateral pins cross each other at the fracture line, satisfactory radiological and clinical results would be achieved with the crossed-pin technique.

Methodology

A total of 59 patients with Gartland extension Type 3 supracondylar humerus fractures who underwent closed reduction and percutaneous crossed-pin fixation were included in the study. K-wires were crossing each other proximal to the fracture site in the proximal crossing group and at the fracture level in the fracture site crossing group. Loss of reduction, Baumann angle, shaft condyle angle, range of motion, and carrying angle were compared between the two groups.

Results

There were 43 males and 16 females in this study, with a mean age of 5.3±2.4 years. The average follow-up duration was 21.9 ± 5.2 weeks. In terms of loss of reduction in the coronal and sagittal planes, there was no statistical difference between the two groups. When the Baumann angle and shaft condylar angle of both groups were analyzed, no statistically significant differences were found at both early postoperative examination and final follow-up.

Conclusions

Although the crossing point of K-wires has been shown to be an important factor in the protection of reduction in biomechanical studies, it was not a significant factor for loss of reduction in this study. Factors except for the crossing point of K-wires may play a more important role in the outcomes of crossed-pin fixation.

## Introduction

Supracondylar humerus fractures occur most often in the first decade of life and account for about 60% of all pediatric elbow fractures [1-3]. Gartland Type 3 fractures involve complete displacement and closed reduction, and percutaneous pin fixation is the standard treatment for these fractures [4,5]. Although it tends to increase iatrogenic ulnar nerve injury, many biomechanical studies have shown that the crossed-pin technique provides superior stability to other pin configurations, including multiple lateral entry pins [6,7].

In the crossed-pin technique for supracondylar humerus fractures, pins should be intraosseous, bicortical, and introduced with the correct size and direction for achieving adequate stability [8,9]. To achieve rotational stability, the correct entry point of pins should be identified by the surgeon so that Kirschner wires (K-wires) would be as far apart as possible when they cross the fracture line and would not cross each other at the fracture level [9]. However, in some circumstances, some of the above principles may not be achievable and, if multiple attempts are made to insert K-wires, the bone may be weakened or the physis of the distal humerus may be damaged. Thus, we designed a retrospective study and hypothesized that even if medial and lateral pins cross each other at the fracture line, satisfactory radiological and clinical results would be achieved with the crossed-pin technique.

In this retrospective clinical study, we compared two groups of children with Gartland Type 3 supracondylar humerus fracture with respect to the crossing point of K-wires in terms of radiological and clinical results after closed reduction and fixation with the crossed-pin technique.

## Materials and methods

The study protocol was approved by the Local Institutional Ethics Committee (KAEK/2020.12.230), and the study was conducted following the principles of the Helsinki Declaration. After approval from the Ethical Committee, a retrospective review of all patients who were treated for supracondylar humerus fracture with crossed-pin fixation between 2015 and 2020 was done from the Hospital Registration System using the International Classification of Diseases code S42.4. A total of 72 patients with Gartland extension Type 3 supracondylar humerus fractures who underwent closed reduction and percutaneous crossed-pin fixation were identified for this study. Among them, 13 patients who were invited for clinical examination but did not come were excluded, and the study was performed among 59 patients. Open fractures, polytrauma patients, flexion-type fractures, patients who underwent open surgery, and patients who were treated with a K-wire combination other than crossed pins were excluded from our study.

**Figure 1:**
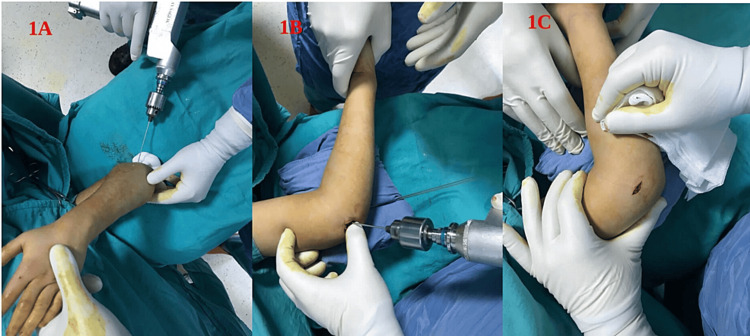
Surgical technique. (A) After reduction, K-wires measuring 2 mm for patients more than six years of age or 1.6 mm for patients six years old or younger were applied for fixation. (B and C) All medial pins were applied in the elbow at less than 80-degree flexion over a small incision centered on the medial epicondyle to prevent surgery-related ulnar nerve injury.

All these cases were treated on an urgent basis rather than an emergent basis and all surgeries were performed 24 hours after trauma. All patients were positioned supine on the operating table and the reduction of the fractures was done by closed reduction. After reduction, K-wires measuring 2 mm for patients more than six years of age or 1.6 mm for patients six years old or younger were applied for fixation (Figure 1A). All medial pins were applied in the elbow at a less than 80-degree flexion over a small incision centered on the medial epicondyle to prevent surgery-related ulnar nerve injury (Figures 1B, 1C). Surgeons considered the Baumann angle, shaft condylar angle, and the intersection between the anterior humeral line and ossification center of capitellum on anteroposterior (AP)/lateral fluoroscopy views as the main determinants of post-fixation reduction quality.

Neurologic and vascular functions were checked postoperatively, and a posterior plaster splint was applied to all patients with the elbow at 90-degree flexion for three to four weeks. After the verification of three cortices bony union, K-wires were removed in the clinic without anesthesia, and the active range of motion was encouraged after the removal of K-wires.

Radiographic evaluation

**Figure 2:**
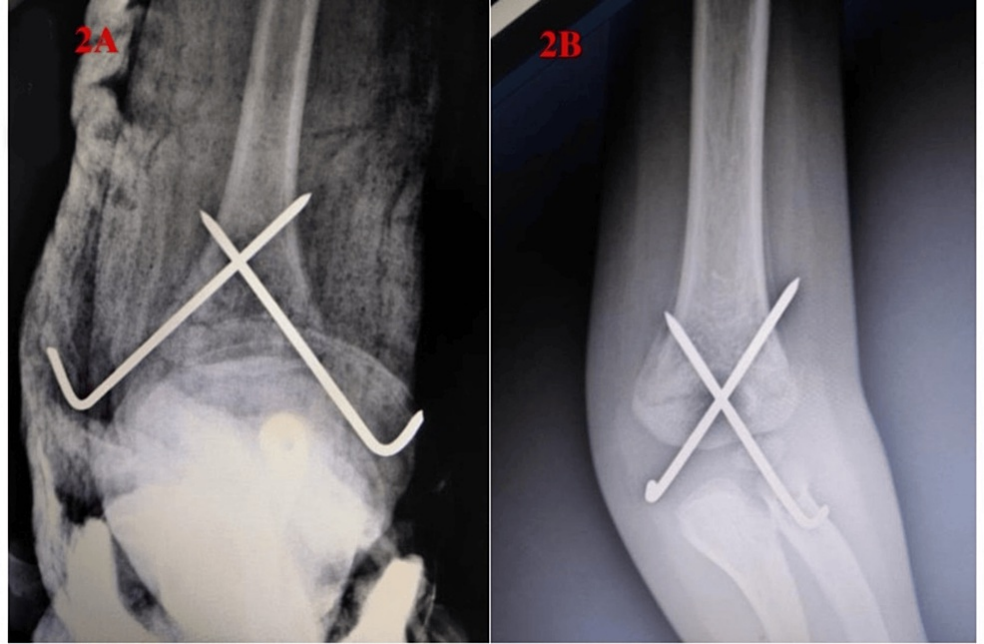
Grouping of patients with respect to the crossing point of K-wires. (A) Crossing of the wire proximal to the fracture line. (B) Crossing of the wire at the fracture line.

A retrospective radiographic review was performed on early postoperative AP/lateral X-rays using hospital radiology records, and patients were grouped with respect to the crossing point of K-wires (Figure 2).

K-wires crossed each other proximal to the fracture site in the proximal crossing (PC) group and at the fracture level in the fracture site crossing (FSC) group. Additionally, early postoperative and last assessment elbow AP/lateral radiographs of all patients were subjected to radiological examination. Moreover, Baumann angles, shaft condyle angles, and the intersection point of the anterior humeral line and ossific nucleus of capitellum were measured by a pediatric orthopedist who was blinded to the study design using hospital radiology records. Loss of reduction was assessed as Kocher et al. and Pennock et al. described in their studies [10,11]. A change of 0-6 degrees in Baumann angle between early postoperative and last assessment AP graphics was described as no displacement, 6-12 degrees as mild displacement, and more than 12 degrees as major displacement. Additionally, a change in shaft condylar angle of 5-10 degrees was described as mild displacement and of 10 degrees as major displacement. Failure of the anterior humeral line to intersect the ossific center of the capitellum was also described as the sagittal plane loss of reduction.

Clinical assessment

**Figure 3:**
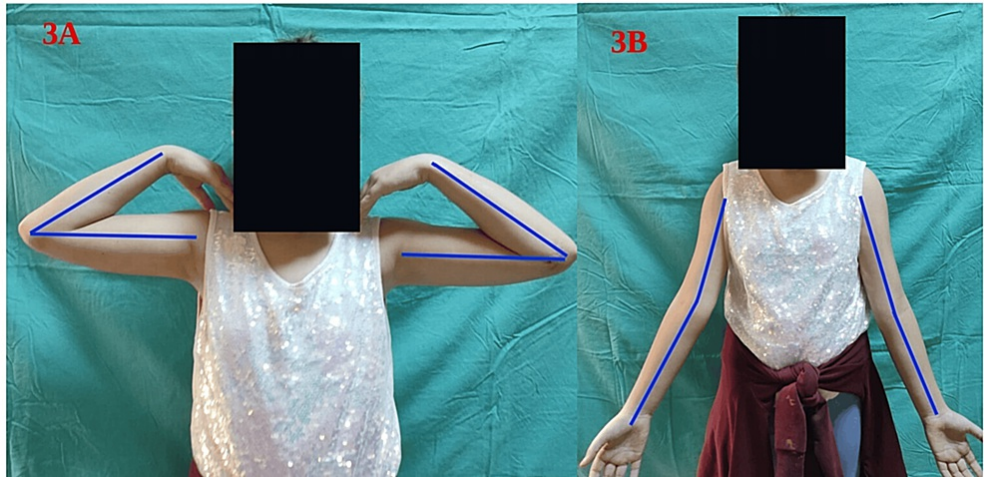
Measurement of the range of motion and carrying angle of both extremities. (A) The range of motion of both extremities of a patient in the study. (B) The carrying angle of both extremities of a patient in the study.

All patients were reached through phone calls and invited for a clinical examination for the measurement of the carrying angle and the range of motion of both extremities (Figure 3).

After comparing both extremities in terms of the range of motion and carrying angle, Flynn criteria [12] (Table 1) were applied to the numerical results. In both groups, patients were classified as excellent, good, fair, and poor regarding functional and cosmetic results. Moreover, surgery-related complications, such as ulnar nerve injury, cubitus varus, and valgus, were examined at the last assessment.

**Table 1:** Flynn criteria.

	Rating	Range of motion	Carrying angle
Satisfactory results	Excellent	0–5	0–5
	Good	>5–10	>5–10
	Fair	>10–15	>10–15
Unsatisfactory results	Poor	>15	>15

Statistical analysis

All statistical analyses were conducted using SPSS version 27 software (IBM Corp., Armonk, NY, USA). Mann-Whitney U test was used for the comparison of independent quantitative variables between the two groups. For the comparison of categorical variables between the two groups, Fisher’s exact test was used. Wilcoxon signed-rank test was used for intragroup comparison of parameters without normal distribution. Comparison of multiple categorical variables between two groups was done using the Fisher-Freeman-Halton exact test using the Monte Carlo Method. P-values <0.05 were described as significant.

## Results

A total of 59 children met the inclusion criteria. K-wires were crossing each other at the fracture site in 30 patients with a mean age of 5.3 years (range = 1-11 years; 22 boys and 8 girls; 16 left and 14 right elbows), whereas the crossing was observed proximal to the fracture site in 29 patients with a mean age of 5.4 years (range = 2-8 years; 21 boys and 8 girls; 18 left and 11 right elbows). The average duration of trauma to surgery was 7.1 hours (range = 3-13 hours). The average follow-up duration was 21.9 weeks. Regarding age, gender, injury site, time until surgery, and follow-up duration, there was no statistical difference between the two groups.

No major displacement was seen in both the groups in the coronal and sagittal planes. Mild displacement in the coronal plane was found in seven (11.8%) patients (two patients in the PC group and five in the FSC group). Further, mild displacement in the sagittal plane was found in four (6.7%) patients (two patients in the PC group and two patients in the FSC group). Regarding loss of reduction in the coronal and sagittal planes, there was no statistical difference between the two groups.

When the Baumann angle and shaft condylar angle of both groups were analyzed, no statistically significant differences were found at both early postoperative examination and final follow-up. Moreover, no significant difference was seen in terms of the change in these angle values between the two groups. In the intragroup comparison of these angles, no significant difference was found between early postoperative examination and final follow-up measurements. Detailed statistical data related to radiological measurements is shown in Table 2.

**Table 2:** Patient data and statistical analysis. *: Mann-Whitney U test. **: Fisher’s exact test. ***: Wilcoxon signed-rank test. ****: Fisher-Freeman-Halton exact test (using the Monte Carlo method).

		Total patients (n = 59)	Crossing proximal to the fracture level (n = 29)	Crossing at the fracture level (n = 30)	P-value
Age (years)	Mean ± SD	5.3 ± 2.4	5.3 ± 1.7	5.3 ± 3.0	0.866^*^
Gender (M:F)	Number	43/16	21/8	22/8	0.937^**^
Injury side (L:R)	Number	34/25	18/11	16/14	0.497^**^
Average time until surgery (hours)	Mean ± SD	7.1 ± 2.2	7.2 ± 2.5	7.1±1.9	0.142^*^
Average follow-up duration (week)	Mean ± SD	21.9 ± 5.2	21.2 ± 4.1	22.5 ± 6	0.626^*^
Loss of reduction (coronal plane)					0.145^****^
No displacement (0–6 degrees)	Number	52 (88.1%)	27 (93.1%)	25 (83.3%)	
Mild displacement (6–12 degrees)	Number	7 (11.8%)	2 (6.8%)	5 (16.6%)	
Major displacement (12 and more)	Number	0	0	0	
Loss of reduction (sagittal plane)					0.887^****^
No displacement	Number	55 (93.2%)	27 (93.1%)	28 (93.3%)	
Mild displacement	Number	4 (6.7%)	2 (6.8%)	2 (6.6%)	
Major displacement	Number	0	0	0	
Baumann angle early postoperative	Mean ± SD	70.3 ± 5.3	70.5 ± 5.2	70 ± 5.4	0.513^*^
Baumann angle last control	Mean ± SD	70.3 ± 5.6	71 ± 4.7	69.6 ± 6.3	0.201^*^
Baumann angle change	Mean ± SD P-value	0.05 ± 3.2	0.3 ± 2.7 0.292^c^	0.4 ± 3.7 0.541^c^	0.331^**^
Shaft condyle angle early postoperative	Mean ± SD	41.2 ± 4.4	40.9 ± 2.2	41.6 ± 5.9	0.843^*^
Shaft condyle angle last control	Mean ± SD	42 ± 4.7	41.5 ± 2.5	42.5 ± 6.1	0.469^*^
Shaft condyle angle change	Mean ± SD P-value	0.8 ± 2.6	0.6 ± 2.7 0.138^***^	0.9 ± 2.5 0.066^***^	0.884^**^

At the final follow-up, the average range of motion was 135.8 degrees and within 6.0 degrees of the opposite elbow in all cases. Furthermore, the average carrying angle was 11.5 degrees and within 6.2 degrees of the opposite elbow in all cases. There was no statistical difference between the two groups regarding the range of motion and carrying angle measurements.

According to the Flynn criteria for range of motion, the results for the PC group were excellent and good in 48.2% each. The result was excellent in 30% and good in 46.6% of the children whose K-wires crossed each other at the fracture site. There was no significant difference between the two groups concerning the Flynn criteria for range of motion. Similarly, no significant difference was found between the two groups concerning the Flynn criteria for carrying angle. The results were excellent in 48.2% and good in 44.8% for the PC group. Meanwhile, the result was excellent in 33.3% and good in 46.6% for the FSC group. Detailed statistical data related to functional and cosmetic results are shown in Table 3, and complications are presented in Table 4.

**Table 3:** Comparison of two groups in terms of Flynn criteria. *: Fisher-Freeman-Halton exact test (using the Monte Carlo method).

	Total patients (n = 59)	Crossing proximal to the fracture level (n = 29)	Crossing at the fracture level (n = 30)	P-value
Flynn grade for range of motion				0.119*
Excellent	23 (38.9%)	14 (48.2%)	9 (30%)	
Good	28 (47.4%)	14 (48.2%)	14 (46.6%)	
Fair	7 (11.8%)	1 (3.4%)	6 (20%)	
Poor	1 (1.6%)	0	1 (3.3%)	
Flynn grade for carrying angle				0.486*
Excellent	24 (40.6%)	14 (48.2%)	10 (33.3%)	
Good	27 (45.7%)	13 (44.8%)	14 (46.6%)	
Fair	5 (8.4%)	1 (3.4%)	4 (13.3%)	
Poor	3 (5%)	1 (3.4%)	2 (6.6%)	

**Table 4:** Complication rates of the patients.

Complications				Outcome
Postoperative ulnar hypoesthesia	2 (3.3%)	1 (3.4%)	1 (3.3%)	Complete recovery
Cubitus varus	1 (1.6%)	0	1 (3.3%)	In observation
Cubitus valgus	2 (3.3%)	1 (3.4%)	1 (3.3%)	In observation
Total	5 (8.4%)	2 (6.8%)	3 (10%)	

Postoperative ulnar hypoesthesia was seen in two patients (one case in each group) and full recovery was obtained in three weeks after surgery for both patients. In two cases, we identified cubitus valgus (one case in each group), and cubitus varus was identified in a case in the FSC group.

## Discussion

The objectives of this study were to evaluate the success rates of our patients who underwent closed reduction and percutaneous crossed-pin fixation for Type 3 supracondylar humerus fracture and review the clinical, radiological, and functional effects of distance between the crossing point of K-wires and the fracture line. In total, according to Flynn criteria, 55 (93.2%) patients had satisfactory results, while four (6.7%) patients were graded unsatisfactory. Of these four patients, two had cubitus valgus, one had cubitus varus, and the remaining patient had a flexion deficit. A meta-analysis by Woratanarat et al. estimated that two cases of loss of fixation and unsatisfactory results would occur for every 100 children treated with the crossed-pin technique [13]. However, the outcomes of the crossed-pin technique varied considerably in previously reported studies. Green et al. described no cases of malunion in their series of 65 patients treated by the crossed-pin technique. Gaston et al. reported 10 malunion cases in 57 children (17.5%), and Maity et al. reported nine cases with malunion in 64 patients (14%) [14-16]. Loss of reduction was noted in 4.2% of 94 patients in a study performed by Pennock et al. [11]. Similarly, this variability between outcomes is the same for ulnar nerve injury after surgery. To prevent this surgery-related complication, we extend the elbow before medial K-wire fixation and make a small incision over the medial epicondyle for direct visualization of the nerve and the entry point of K-wires. Postoperative ulnar hypoesthesia was seen in two patients and both recovered three weeks after surgery. However, despite these measures taken by the surgeons, there is an increased risk of ulnar nerve injury with crossed-pin fixation. Slobogean et al. showed that one case of ulnar nerve injury would occur for every 28 children (3.5%) treated with the crossed-pin technique [17]. In a meta-analysis performed by Dekker, this risk occurs in one for every 27 children (3.7%) [18].

We believe that this variability between outcomes of crossed-pin groups has its source in multiple technical requirements that should be followed by surgeons to achieve satisfactory results with the crossed-pin technique. A clinical study that investigated the reasons for the loss of fixation performed by Sankar et al. showed that failure to engage both fragments, failure to achieve bicortical fixation, and failure to achieve adequate pin separation at the fracture site are the most common errors [8]. Pennock et al. showed that the only significant factor associated with loss of reduction was pin spread in a study performed among children treated with lateral pin configuration [11]. In a biomechanical study, Gottschalk et al. found out that the starting point and pin size affect the stability of fixation [9].

In our study, among those technical errors, we aimed to dwell on the crossing point of K-wires. At first glance, it may seem quite striking that 50% of this cohort ended up with the crossing point at the fracture level. However, in clinical practice, surgeons want to complete these surgeries with the fewest number of pin application attempts because every attempt increases the risk of ulnar nerve injury and damage to the distal humerus physis. Hence, although crossing pins at the fracture site has been considered a technical error to be avoided, in some cases, surgeons show an unwillingness to revise K-wires that cross each other at the fracture site to avoid multiple attempts. Some surgeons believe that they can manage this issue with close clinic observation. As the number of pin application attempts was not recorded, we were unable to present the number of attempts made by the surgeons to achieve more conformity to technical recommendations in this study.

In this study, no significant difference was found related to the crossing point of K-wires in the radiological and clinical comparisons of both groups. However, when the data obtained is examined in detail, the functional and cosmetic results according to Flynn criteria are more satisfactory in the PC group compared to the FSC group. The number of patients with fair and poor results was higher in the FSC group than that in the PC group. Fair results regarding the range of motion were detected six times more in the FSC group compared to the PC group. Moreover, fair results were detected four times more in the FSC group than in the PC group cosmetically. Furthermore, the number of excellent cases was lower in the FSC group than in the PC group. However, a major loss of reduction was not detected in the FSC group, and outcomes related to loss of reduction were similar to the PC group. Hence, in our opinion, some other factors such as the entry point of K-wires or medial wall comminution may play a more important role than the crossing point of K-wires in functional and cosmetic outcomes. Overall, this may be due to the increased incidence of other technical errors seen when K-wires cross each other at the fracture site.

Although no major displacement was seen in both groups, we observed malunion in three cases. We investigated this discrepancy and found out that the low quality of initial fixation was the reason for this result. To reduce radiation exposure, X-rays of the uninjured elbows of patients had not been taken after surgery. Hence, in our study, we were not able to evaluate post-surgery fixation quality and were not able to exclude patients without acceptable reduction. According to Gaston et al., malreduced fractures must be excluded from studies to differentiate fractures pinned in the anatomic position [15]. Therefore, despite the lack of major displacement seen in both groups, we observed elbow deformity in three patients. We examined the radiology records of these patients in detail and observed some technical errors such as improper pin entry point or lack of engagement of both fragments and fracture-related factors such as medial and lateral condylar comminution.

The main limitation of this study was its retrospective design. No power analysis was done in the study. Moreover, all radiologic measurements were performed by an orthopedic surgeon, and intraobserver and interobserver validity and reliability of the measurements were not examined. Additionally, the present study did not exclude patients with unacceptable reduction.

## Conclusions

The crossing-pin technique appears to be effective in the treatment of Type 3 supracondylar humerus fractures. Although the crossing point of K-wires has been shown to be an important factor in the protection of reduction in biomechanical studies, it was not a significant factor for loss of reduction in this study. Factors except for the crossing point of K-wires may play a more important role in the outcomes of crossed-pin fixation. Prospective studies focusing on the reasons for unsatisfactory outcomes after crossed-pin fixation should be conducted in the future.
